# Preserving Porcine Genetics: A Simple and Effective Method for On-Site Cryopreservation of Ear Tissue Using Direct Cover Vitrification

**DOI:** 10.3390/ijms24087469

**Published:** 2023-04-18

**Authors:** Xia Zhang, Xin Liu, Xiao-Li Liu, Dan-Ya Wu, Kai Zhou, Zhi-Sheng Yu, Cheng-Li Dou, Tian Xu, Mei Yu, Yi-Liang Miao

**Affiliations:** 1 Institute of Stem Cell and Regenerative Biology, College of Animal Science and Veterinary Medicine, Huazhong Agricultural University, Wuhan 430070, China; 2 Key Laboratory of Agricultural Animal Genetics, Breeding and Reproduction, Huazhong Agricultural University, Ministry of Education, Wuhan 430070, China; 3 National Demonstration Center for Experimental Veterinary Medicine Education, Huazhong Agricultural University, Wuhan 430070, China; 4 Frontiers Science Center for Animal Breeding and Sustainable Production, Huazhong Agricultural University, Ministry of Education, Wuhan 430070, China

**Keywords:** pig, vitrification, genetic conservation, ear tissue

## Abstract

Cell cryopreservation is widely used for porcine genetic conservation; however, isolating and freezing primary cells in farms without adequate experimental equipment and environment poses a significant challenge. Therefore, it is necessary to establish a quick and simple method to freeze tissues on-site, which can be used for deriving primary fibroblasts when needed to achieve porcine genetic conservation. In this study, we explored a suitable approach for porcine ear tissue cryopreservation. The porcine ear tissues were cut into strips and frozen by direct cover vitrification (DCV) in the cryoprotectant solution with 15% EG, 15% DMSO and 0.1 M trehalose. Histological analysis and ultrastructural evaluation revealed that thawed tissues had normal tissue structure. More importantly, viable fibroblasts could be derived from these tissues frozen in liquid nitrogen for up to 6 months. Cells derived from thawed tissues did not show any cell apoptosis, had normal karyotypes and could be used for nuclear transfer. These results suggest that this quick and simple ear tissue cryopreservation method can be applied for porcine genetic conservation, especially in the face of a deadly emerging disease in pigs.

## 1. Introduction

The outbreak of deadly Africa Swine Fever (ASF) brings a great threat to the production and conservation of pig germplasm resources, and it is crucial to protect these resources effectively. Currently, the main preservation methods are semen or somatic cell cryopreservation in pigs. For semen cryopreservation, it can only preserve half of the porcine genetic information. By contrast, somatic cell cryopreservation becomes the best way to preserve the whole of porcine genetic information. However, somatic cell cryopreservation needs to transport pig tissues to the cell culture room for primary cell isolation, culture and freezing, which is not feasible to perform directly on pig farms. Therefore, it is necessary to establish a method to freeze ear tissues for porcine genetic conservation that is quick, simple, low-cost and can be performed on-site, especially during regional lockdowns when pig epidemic diseases break out.

Animal tissues can be stored at −20 °C, −80 °C and −196 °C with or without cryoprotectant. However, the viability of deriving the primary cells declines with the increase in storage time. It is shown that somatic cells can be isolated and cultured from auricular cartilage and muscle tissues stored at 4 °C for 216 h [1]. If animal bodies were kept frozen at −20 °C for up to 16 years without any cryoprotection, the cells were dead after thawing [2]. Zhang et al. reported that they failed to isolate and culture cells from frozen boar ears without cryoprotectant at −20 °C stored for 3 years. By contrast, if these ears were stored at −80 °C or −196 °C for 3 years without cryoprotectant, live cells could be isolated and cultured. Unfortunately, when these cells were used as donor cells for nuclear transfer, the blastocyst formation rate of cloned embryos decreased significantly when compared to fresh ears [3]. Thus, the cryoprotectant is crucial for pig germplasm resource conservation, especially for the long-term storage of animal tissues.

Cryoprotectants include permeable cryoprotectants, such as dimethyl sulfoxide (DMSO), ethylene glycol (EG) and non-permeable cryoprotectants, like sucrose. Permeable cryoprotectants are low molecular weight chemicals and can penetrate the cell membrane and replace the water in the cytoplasm. Non-permeable cryoprotectants are high molecular weight agents and cannot enter cells but form a hypertonic environment around the cells. Trehalose is a non-reducing disaccharide composed of two glucose molecules and is widely used in the cryopreservation of cells and tissues. Trehalose can be used as a substitute for sucrose and has been shown to protect cells’ integrity against stresses such as low temperature and oxidation. However, little is known about the effects of trehalose on the frozen-thawed process of porcine ear tissue.

In this study, we integrated the present animal tissue cryopreservation methods and developed a simple method for rapidly freezing porcine ear tissue and detected the efficiency of primary fibroblast isolation and proliferation after thawing the frozen ear tissue. We intend to apply this method to somatic cell preservation in local farm-raised and newly-cultivated pig breeds. In the event of an epidemic disease outbreak threatening and eliminating these pigs, we can reproduce the local or newly cultivated pigs through somatic cell nuclear transfer (SCNT) technology. Therefore, our studies have established a method to freeze ear tissues for porcine genetic conservation that Is quick, simple, low-cost and can be performed on-site. Additionally, this method can be used for wildlife resource conservation. 

## 2. Results

### 2.1. Effects of Different Vitrification Methods on Cell Viability from Thawed Ear Tissues

**Figure 1:**
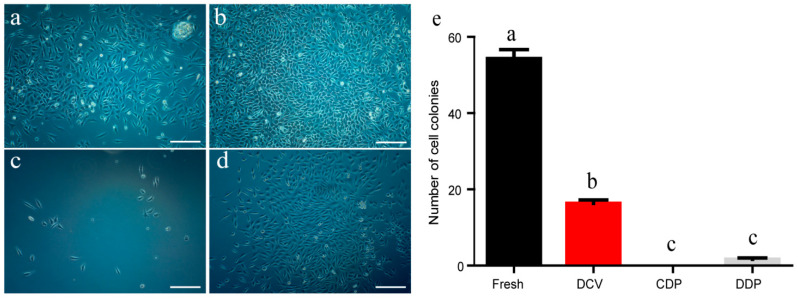
Effects of different vitrification methods on cell viability from thawed ear tissues. (**a**) Cells isolated from fresh tissue; (**b**) Cells isolated from tissues frozen by direct cover vitrification (DCV) method; (**c**) Cells isolated from tissues by cryovials directly plunged into LN2 (CDP) method; (**d**) Cells isolated from tissues by droplet directly plunged into LN2 (DDP) method; (**e**) Number of cell colonies with different vitrification methods. Error bars represent the SEM. Scale bar = 200 µm.

To evaluate the efficiency of different vitrification methods, we isolated cells from thawed tissues that had been frozen for 24 h and evaluated their viabilities to derive primary fibroblast colonies (Figure 1). The vitrification solution used was PBS with 20% FBS + 15% EG + 15% DMSO + 0.5 M sucrose. We found that viable cells could be isolated from each group (Figure 1a–d). The results showed that the direct cover vitrification (DCV) group had the highest number of cell colonies compared with other vitrification groups (Figure 1e). In contrast, although primary cells could grow using the frozen tissues from cryovials directly plunged into LN2 (CDP) group, there were no colonies formed (Figure 1e). Furthermore, the droplets directly plunged into LN2 (DDP) group had cell colony formation, but the number of colonies was lower than the DCV group (Figure 1e). Therefore, we used the DCV method to perform porcine ear tissue cryopreservation in the following experiment.

### 2.2. Effects of Tissue Size on the Vitrification of Ear Tissues

**Figure 2:**
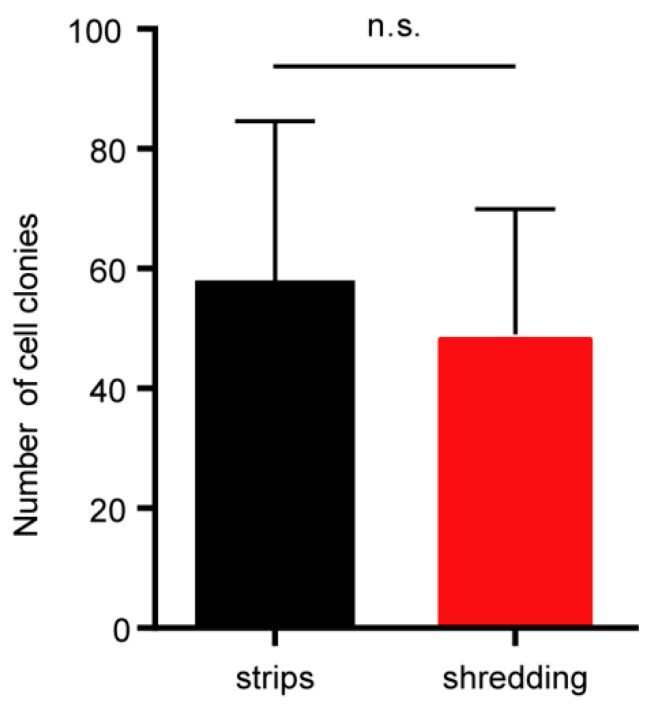
Effects of tissue size on the vitrification of ear tissues. Numbers of cell colonies obtained from different sizes of frozen-thawed tissue. Error bars represent the SEM.

More research has been done to discover whether tissue size affects how effectively frozen tissue may produce primary fibroblasts. We cut the porcine ear tissues into small pieces with scissors before freezing and exposed them fully to cryoprotective solution. However, this method takes more time and becomes unprotectable from microbial contamination when this method is operated on-site at pig farms. To simplify the pretreatment of ear tissues before freezing, we tried to cut the ear tissues into strips (5 mm × 1 mm × 1 mm) before freezing. The results showed that cells could easily grow from these thawed strips, with more than 50 cell colonies formed. The efficiency of frozen tissue strips was comparable with the conventional method of getting primary cells from shredded frozen tissues (Figure 2).

### 2.3. Effects of Different Cryoprotectants on the Vitrification of Ear Tissues

**Figure 3:**
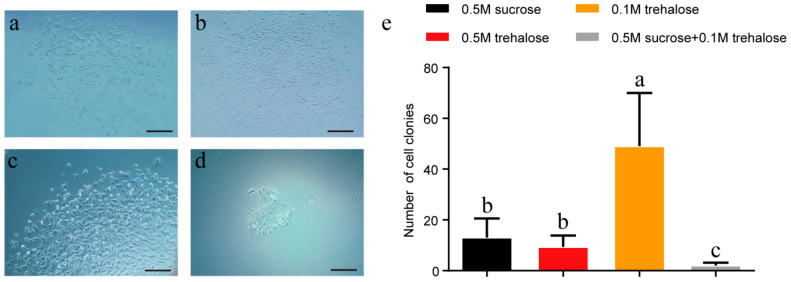
Effects of trehalose on the vitrification of ear tissues. (**a**) Cell derivation from tissues frozen using vitrification solution with 0.5 M sucrose; (**b**) Cell derivation from tissues frozen using vitrification solution with 0.5 M trehalose; (**c**) Cell derivation from tissues frozen using vitrification solution with 0.1 M trehalose; (**d**) Cell derivation from tissues frozen using vitrification solution with 0.5 M trehalose and 0.1 M trehalose; (**e**) Numbers of cell colonies with different vitrification solutions. Error bars represent the SEM. Scale bar = 200 µm.

To evaluate the effects of different cryoprotectants on the vitrification of porcine ear tissues using the DCV method, we used PBS containing 20% FBS, 15% EG, 15% DMSO and either 0.5 M sucrose, 0.5 M trehalose, 0.1 M trehalose, or a combination of 0.5 M sucrose and 0.1 M trehalose to freeze tissue strips. After freezing these ear tissues for 24 h, we thawed the tissues and performed primary cell culture. Here, we found that viable cells could be isolated from each group (Figure 3a–d). Unexpectedly, the group treated with 0.1 M trehalose had significantly higher cell colony numbers than the other groups. (Figure 3e). Therefore, we added 0.1 M trehalose to the cryoprotectant solution in the following experiments.

### 2.4. Histological Analysis of Cryopreserved Ear Tissues

**Figure 4:**
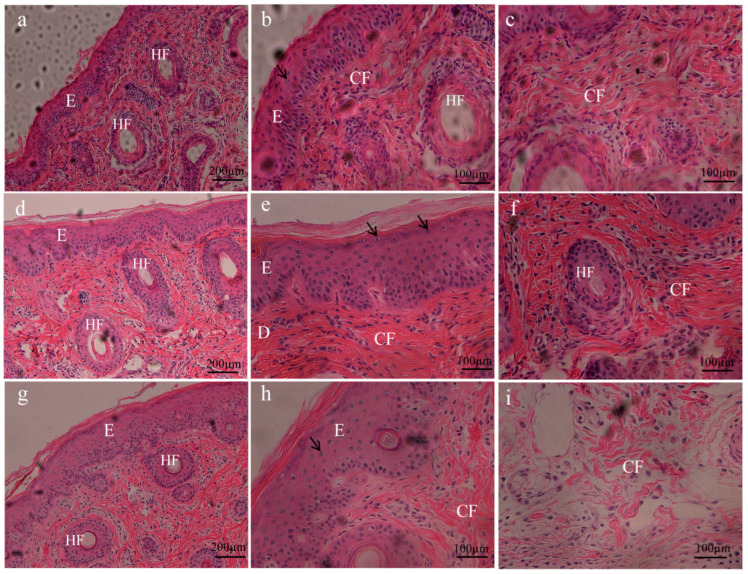
The histological analysis of ear tissues cryopreserved for 6 months. HE staining in representative pig ear sections obtained from fresh tissue, cryopreserved tissue and the tissue frozen without protection. (**a**–**c**): fresh tissues; (**d**–**f**): cryopreserved tissues; (**g**–**i**): tissue frozen without protection. E: Epidermis, D: Dermis, CF: Collagen fiber, HF: Hair follicle. ↑: epidermal cell. Scale bar = 200 µm in (**a**,**d**,**g**); Scale bar = 100 µm in (**b**,**c**,**e**,**f**,**h**,**i**).

To examine the effects of our cryopreservation method on the tissue structure and morphology, we analyzed sections from cryopreserved ear tissues frozen for 6 months and fresh tissues using histological analysis. Ear tissues frozen without protection served as the negative control. As shown in Figure 4a,b, the epidermal cell structure in fresh tissues was clear and arranged neatly and evenly, with round or oval nuclei. In the negative control group, epidermal nuclei were lightly stained, and there were cavities in tissues (Figure 4g,h). However, the cell structure was clear and arranged neatly in the frozen group with cryoprotection (Figure 4d,e). In the dermis area, the collagen fibers in fresh tissue were arranged in order in the longitudinal section, and the fiber bundles were arranged compactly in the transverse section (Figure 4c). The fiber bundles of the unprotected frozen group were loosely arranged, and there were many fractures (Figure 4i). However, tissue structure in the frozen group with cryoprotection was similar to that of fresh tissues (Figure 4f).

### 2.5. Ultrastructural Evaluation of Cryopreserved Ear Tissues

**Figure 5:**
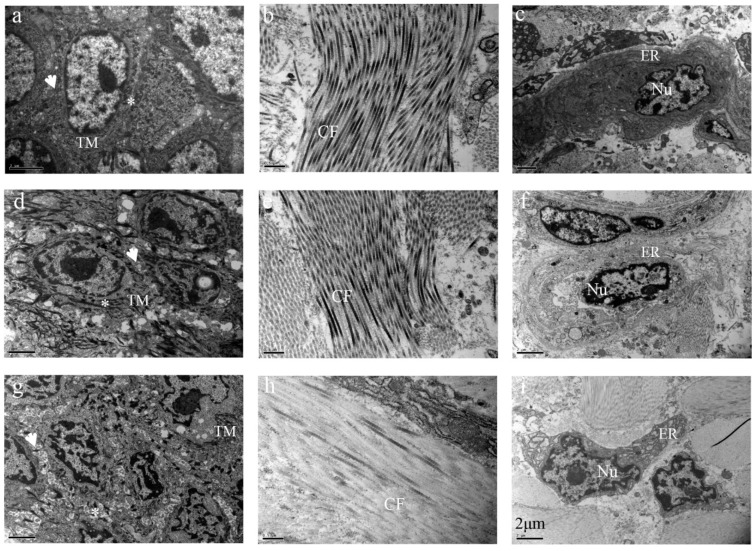
Ultrastructural evaluation of ear tissues cryopreserved for 6 months. (**a**–**c**), fresh tissue; (**d**–**f**), cryopreserved tissue; (**g**–**i**), frozen tissue without protection. Nu: Nuclear; CF: Collagen fibers; ER: Endoplasmic reticulum; TM: Tension micro-filaments; *: Desmosomes; ↑: intercellular space.

It was well known that ultrastructure in cells and tissues was sensitive to the drastic change in temperature. To evaluate the effects of vitrification on the ear tissues, we used transmission electron microscopy to observe the thawed tissues. As shown in Figure 5a,d, there were many desmosomes around spine cells in fresh and frozen with protection groups and a large number of tension microfilaments in the cytoplasm. In the unprotected frozen group, the number of desmosomes and tension decreased significantly, and intercellular space increased significantly (Figure 5g). In the dermis, collagen fibers in fresh and frozen with protection groups had light and dark periodic striations (Figure 5b,e). In the unprotected frozen group, periodic striations on the collagen fibers disappeared, boundaries between the collagen fibers were not clear, and electron density was decreased (Figure 5h). Moreover, the endoplasmic reticulum of fibroblasts was expanded and vacuolated in the unprotected frozen group (Figure 5i). Nevertheless, when compared to the fresh group, the electron density of the fibroblast’s cytoplasm was decreased in both the protected and unprotected frozen groups (Figure 5f,i).

### 2.6. Effects of Cryopreserved Time on the Vitrification of Ear Tissues

**Figure 6:**
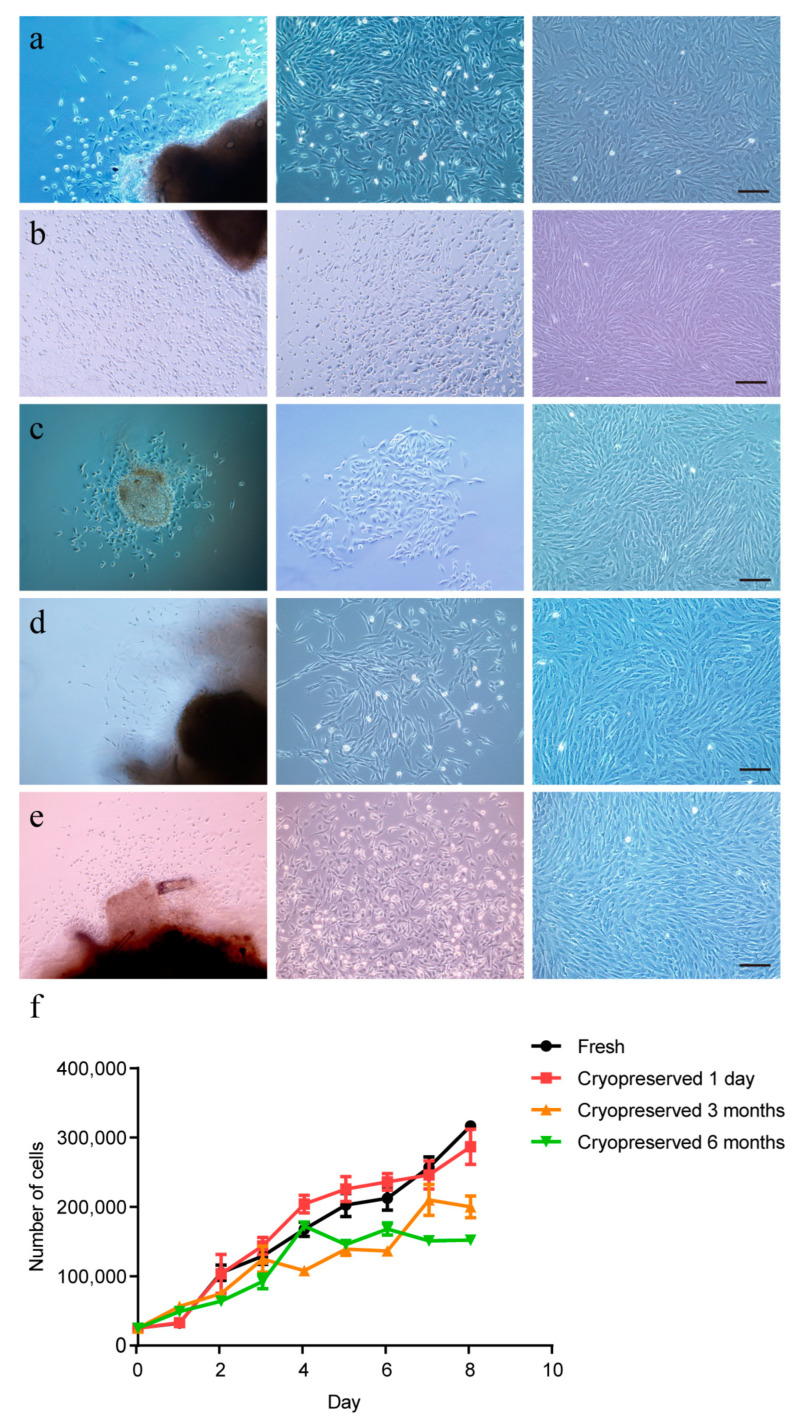
Effects of cryopreserved time on the vitrification of ear tissues. Viable cells isolated from porcine ear tissues cryopreserved for (**a**) 1 day, (**b**) 1 week, (**c**) 1 month, (**d**) 3 months and (**e**) 6 months. (**f**) Cell growth curves for the cells isolated from fresh tissues and frozen-thawed tissues cryopreserved for 1 day, 3 months and 6 months. The error bars represent the SEM. Scale bar = 200 µm.

To detect the effects of cryopreserved time on cell isolation efficiency and viability, we performed primary fibroblast culture from thawed tissues that were cryopreserved for 1 day, 1 week, 1 month, 3 months and 6 months. The results showed fibroblasts could be isolated from all thawed tissues cryopreserved for different times (Figure 6a–e). Furthermore, these primary cells could be passaged normally (Figure 6a–e). To compare the growth vitality of different groups, we delineated the cell growth curve of fibroblasts derived from fresh tissue and thawed tissues cryopreserved for 1 day, 3 months and 6 months (Figure 6f). The results showed that the growth trends of cells in the fresh group and the frozen group were similar. However, the growth trends in the 3-month cryopreserved group and the 6-month cryopreserved group decreased with the extension of culture time.

### 2.7. Effects of Ear Tissue Vitrification on the Apoptosis and Karyotype of Primary Cells

**Figure 7:**
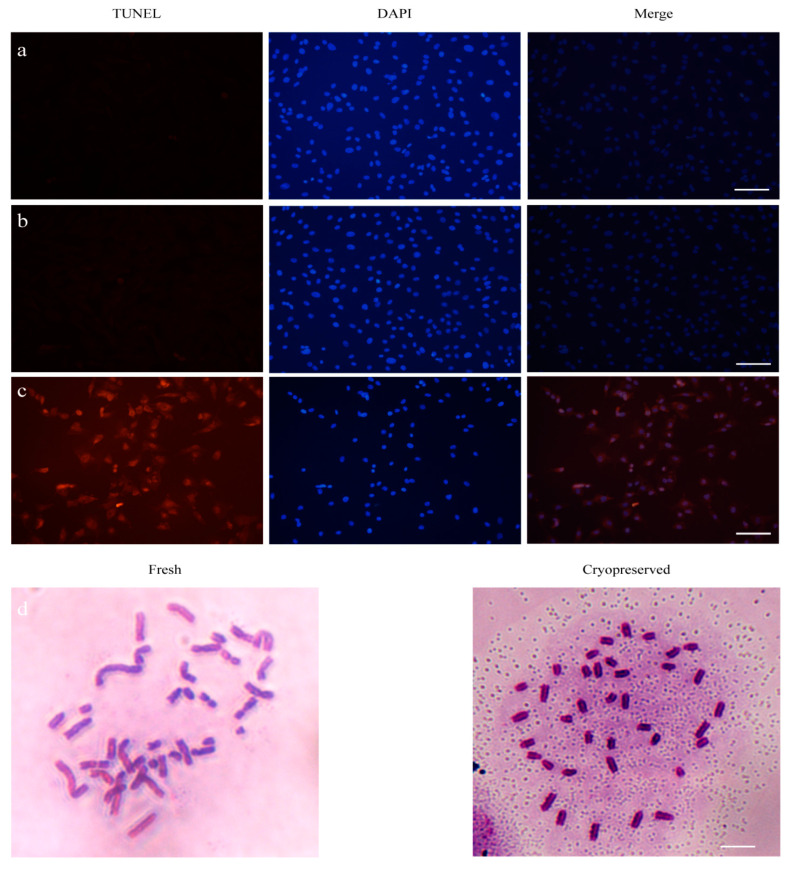
Effects of ear tissue vitrification on cell apoptosis and karyotype. Representative images of cells isolated from fresh tissues and cryopreserved tissues for TUNEL assay: (**a**) cells isolated from fresh tissues; (**b**) cells isolated from thawed tissues; (**c**) H_2_O_2_ treated group. Scale bar = 200 µm; (**d**) Representative images of cell karyotype, Scale bar = 50 µm.

Genome stability is critical for genetic conservation and animal cloning through SCNT technology. To study the effects of ear tissue vitrification on this test index, we detected the apoptotic cell signal and the karyotype of fibroblasts which were isolated from 6-month cryopreserved tissue. Through the TUNEL assay, cells isolated from fresh tissue and treated with H_2_O_2_ were used as negative and positive controls, respectively. The result showed that cells derived from fresh tissue and cryopreserved tissue did not show any cell apoptotic signal (Figure 7a–c). Furthermore, we also detected the karyotype of cells derived from fresh and cryopreserved tissue. The results showed that there was no difference between the two origins of primary porcine fibroblasts, and both groups have 38 chromosomes (Figure 7d). These results suggested that our quick and simple cryopreservation strategy could keep the complete genome stability of porcine ear tissues.

### 2.8. Effects of Ear Tissue Vitrification on the Development of SCNT Embryos

**Figure 8:**
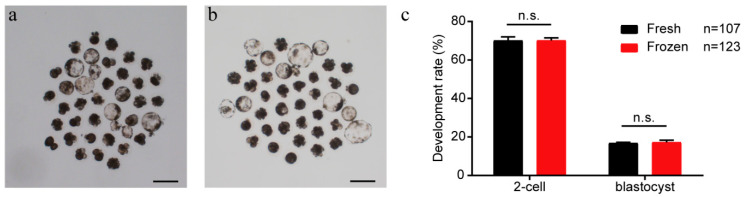
Effects of ear tissue vitrification on the development of SCNT embryos. (**a**,**b**) Representative images of SCNT blastocysts constructed by using cells isolated from fresh (**a**) and thawed (**b**) tissues. Scale bar = 200 µm; (**c**) Percentages of cleavage and blastocyst for SCNT embryos. Error bars represent the SEM.

The final aim of tissue cryopreservation or cell cryopreservation is to reproduce full-term developed animals with their original genetic material through SCNT technology. So, we need to examine the development rates of SCNT embryos using primary fibroblasts derived from frozen tissues. Here, we compared the developmental competencies of SCNT embryos using the donor cells derived from fresh tissues and 6-month cryopreserved tissues. We reconstructed 107 SCNT embryos using donor cells derived from fresh tissues and 123 SCNT embryos using donor cells derived from 6-month cryopreserved tissues. We compared the percentages of cleavage and blastocyst formation in both groups and found no significant differences (Figure 8a–c), indicating that our cryopreservation strategy did not affect the developmental competence of donor fibroblasts for porcine SCNT embryos. Therefore, our results suggested that ear tissue vitrification is an efficient and reliable method for achieving porcine genetic conservation through SCNT technology.

## 3. Discussion

The traditional method of somatic cell cryopreservation requires fresh tissue to be collected and transported immediately to a lab equipped with a clean and complete cell culture room for cell isolation, culture and subsequent cryopreservation. However, this method is not always feasible, particularly during disease outbreaks when pig farms may be placed under lockdown and cannot operate in time for cell isolation. In such situations, on-site tissue cryopreservation can be a viable alternative. As pig ear tissue is a kind of multicellular tissue, the penetration rate of cryoprotectant to the ear tissue is slower than that of the single cell. Using two or three cryoprotectants simultaneously has been shown to reduce toxicity and increase the efficiency of cryopreservation [4], thereby enhancing cell viability.

In this study, we found that the DCV method obtained the highest number of cell colonies among these three methods, and it was able to maintain the normal morphology of the cryopreserved tissue to a greater extent. This can be attributed to the fact that the DCV method allows for rapid cooling, which promotes vitrification and reduces ice crystal formation [5]. During the thawing process, the vitrified tissues were directly placed into the thawing solution to maximize the warming rate and minimize devitrification. Yeoman et al. performed vitrification of cortical pieces of monkey ovarian tissues by the DDP method [6]. The larger volume of vitrification solution around the tissue pieces and the formation of nitrogen vapor might slow down the cooling rate of LN2 and reduce the heating rate during thawing. Dropping the individual strips in liquid nitrogen could be time-consuming and might increase the exposure time of tissues to dehydrating solution [7]. Similarly, the CDP method also resulted in a slower cooling rate of LN2, which can be a limitation of this approach.

Trehalose is a non-permeable cryoprotectant that has been shown to have beneficial effects in cryopreservation. Brito et al. demonstrated that trehalose could maintain the numbers of normal follicles during cryopreservation of ovarian tissue [8]. Erdag et al. also found that adding trehalose to the cryopreservation solution can improve cell membrane integrity by up to 65% [9]. Trehalose can protect cells or tissues in a variety of ways and protect cell integrity from environmental pressures, such as dehydration, cold and oxidation [10]. Brito et al. indicated that trehalose prevented osmotic stress better than sucrose [8]. It was previously shown that 0.5 M sucrose was of no benefit during the vitrification of feline ovarian tissue [11] and bovine ovarian tissue [12]. This is similar to our result that adding 0.1 M trehalose to the cryoprotectants could get better-frozen results than using 0.5 M sucrose and 0.5 M trehalose. In the group supplemented with both 0.5 M sucrose and 0.1 M trehalose, the number of cell colonies was the lowest, which may be due to the interference of 0.5 M sucrose with the ability of trehalose to prevent osmotic stress. Moreover, the key to vitrification is to make cryoprotective agents penetrate into tissues quickly, and effective tissue penetration is the prerequisite for the tissue to enter the vitrification state smoothly during the cooling process [13]. Theoretically, the smaller the tissue, the better the penetration effect of cryoprotective agents. However, using larger tissue can simplify the pretreatment procedure before freezing and save time. Gook et al. showed that trimming ovarian tissue to 2.0–5.0 mm would not affect the cryopreservation effect, and a large number of preantral follicles could be protected [14]. In this study, we found that strips (5 mm × 1 mm × 1 mm) of porcine ear tissues could be frozen successfully and showed no significant detrimental effects on the isolate of primary fibroblasts.

In our study, we observed that thawed tissues without cryoprotection exhibited a significant loss of desmosomes, a larger gap between cells and damage to the structure of collagen fibers. However, the DCV group with cryoprotection reduced these injuries. Porcine ear tissues have a large number of spinous cells in the epidermal layer, with large nuclei, fine-grained chromatin and abundant tension filaments and mitochondria in the cytoplasm. We also observed that small protrusions protruded around cells and connected with adjacent protrusions to form desmosomes. Desmosomes are a unique intercellular junction structure of epithelial cells, which play a role in the connection between cells. For tissues, the anchoring of intercellular connections to cells further strengthens the fixation of the cytoskeleton on cells and makes their movement and shape changes more limited, so they are more sensitive to the freezing process. During the freezing process of cells, cell volumes change because of the continuous crystallization of extracellular water and the increase of solute concentration. If the cooling speed is too fast, water has no time to exude or exudes rapidly, and the cell membrane has insufficient ability to adapt to this volume change, resulting in cell rupture, which will cause cell damage or even death. Additionally, the presence of tight junctions can decrease cell compliance, making it more difficult for the cell membrane to adapt to volume changes and leading to damage [15]. Ferreira et al. found that cryopreservation decreased the number of desmosomes in horse embryos [16]. In this study, the cryoprotectant combination of DMSO, EG and trehalose reduced the damage to the cell membrane during cryopreservation, preventing significant loss of desmosomes. Concerning nuclear abnormalities deriving from cryopreservation, aneuploidy and chromosome breakage in human fibroblasts has been observed [17]; by contrast, the apoptosis and karyotype of porcine fibroblasts obtained using our strategy have no abnormalities.

In conclusion, we established a quick and simple method to freeze ear tissues for porcine genetic conservation. The method involves cutting the ear tissues into 5 mm × 1 mm × 1 mm strips and using direct cover vitrification (DCV) with a cryoprotectant solution containing 0.1 M trehalose for a minimum of 6 months. Histological analysis and ultrastructural evaluation showed that the thawed tissue maintained normal tissue structure and stable genetic material. More importantly, fibroblasts derived from the thawed tissues could be used for nuclear transfer to generate cloned pigs.

## 4. Materials and Methods

Chemicals and reagents used in the present study were purchased from Sigma Chemical Co. (Beijing, China) unless otherwise specified. The experiments were conducted following the guidance of the Animal Care and Use Committee of Huazhong Agricultural University (Ethical number: HZAUSW-2021-0049).

### 4.1. Freezing and Thawing of Ear Tissues

The experimental pigs were obtained from the pig farm of Huazhong Agricultural University, and a total of 9 pigs were used in this study. The ear tissues were washed in 70% ethanol and PBS with a 2% antibiotic-antimycotic solution.

According to the requirements of different experiments, ear tissues were then shredded or cut into strips (5 mm × 1 mm × 1 mm) and pretreated with an equilibration solution composed of 7.5% (*v*/*v*) ethylene glycol (EG) and 7.5% (*v*/*v*) dimethylsulphoxide (DMSO) in DPBS with 20% fetal bovine serum (FBS) for 5 min at room temperature. They were transferred to a vitrification solution composed of 15% EG and 15% DMSO and sucrose or trehalose for 5 min at room temperature. After treatment, the tissues were frozen by different methods.

Method 1: We modified the direct cover vitrification (DVC) procedure of Chen et al. [5]. The pretreated ear tissues were placed on a piece of gauze to remove the surrounding vitrification solution. The tissues were then put into a 1.8 mL plastic cryovial. Liquid nitrogen (LN2) was applied to the ear tissues for vitrification. Then the cap of the cryovial was closed and placed into an LN2 tank.

Method 2: Cryovial was directly plunged into LN2 (CDP): After dehydration, the tissues were placed in a 1.8 mL plastic cryovial containing 50 µL vitrification solution. Then the cap of the cryovial was closed and placed into an LN2 tank.

Method 3: Droplets were directly plunged into LN2 (DDP): After dehydration, the tissues were immediately drawn into a Pasteur pipette. Individual drops were released into a shallow container of LN2 gently. These solid drops were then collected with precooled forceps, sealed in LN2-filled cryovials and stored in LN2.

For tissue thawing, the cryovial was removed from the LN2 and its cap was opened. After volatilizing LN2, ear tissues were transferred to a 1 mL thawing solution consisting of 0.5 M sucrose or 0.1 M trehalose and 20% FBS and kept for 5 min at room temperature. Then the tissues were transferred to 0.25 M sucrose or 0.05 M trehalose with 20% FBS for 5 min each. The ear tissues were then subjected to detection or cell isolation.

### 4.2. Histological Evaluation

Ear tissues frozen for 6 months with or without cryopreserved were fixed with 4% paraformaldehyde (PFA) and embedded in paraffin. Fresh tissues were used as controls. Histological slides were prepared according to standard operating procedures, and the slides were stained with H&E for morphological examination [18].

### 4.3. Ultrastructural Evaluation

After thawing, the ear tissues were prepared for ultrastructural evaluation. Fresh tissues were used as controls. The thawed tissues were fixed in 2% glutaraldehyde in 0.1 M phosphate buffer (pH 7.4) for 1–7 days. The procedure was modified as described previously [19]. After being rinsed 3 times in a 0.1 M sodium phosphate buffer (pH 7.0) at room temperature, tissues were post-fixed with 2% osmium tetroxide in PBS. Then, tissues were dehydrated in a graded series of acetone, infiltrated and embedded in epoxy resin SPI-812. Ultra-thin sections (60–80 nm) were cut with a diamond knife and an ultramicrotome, mounted on slotted and formvar-coated grids and stained with 5% uranyl acetate along with 5% lead citrate. These sections were examined and imaged using HITACHI transmission electron microscopy (TEM).

### 4.4. Somatic Cell Isolation

Thawed ear tissues were cut into pieces, then digested with 0.05% trypsin at 37 °C for 30 min. The digestion was terminated by DMEM with 15% FBS. Digested tissues were pelleted and resuspended in a DMEM medium consisting of 15% FBS and 1% antibiotic-antimycotic solution. The fibroblasts were then plated in a 6-well plate and cultured at 38.5 °C and 5% CO_2_ in a humidified atmosphere. The culture medium was changed every other day.

### 4.5. Somatic Cell Nuclear Transfer (SCNT)

The SCNT protocol used in this study was modified as described previously [20]. Porcine ovaries were collected from a local slaughterhouse. Cumulus oocyte complexes (COCs) were aspirated from 3–6 mm antral follicles. Approximately 30 COCs per well were cultured in a 96-well plate. After 40–42 h, cumulus cells were removed from the oocyte by vortexing for 5 min in 0.1% bovine testicular hyaluronidase. Oocytes were incubated in manipulation media (Ca-free NCSU-23 with 5% FBS) containing 7.5 μg/mL cytochalasin B for 5 min. Following the incubation period, oocytes were enucleated by removing the first polar body and metaphase II plate. A single fibroblast from ear tissue was injected and fused to the enucleated oocyte. Fusion/Activation was induced by 2 DC pulses of 140 V for 40 μsec in 280 mM mannitol, 0.001 mM CaCl_2_ and 0.05 mM MgCl_2_. After fusion/activation by electroporation, the reconstructed embryos (day 0) were cultured in a PZM3 medium at 38.5 °C and 5% CO_2_ in a humidified atmosphere until the blastocyst stage (day 7).

### 4.6. Terminal Deoxynucleotidyl Transferase Assay (TUNEL)

TUNEL staining was carried out using the One Step TUNEL Apoptosis Assay Kit (Beyotime Biotechnology) following the manufacturer’s instructions. Cells isolated from cryopreserved tissue were cultured and then put through screening. Cells treated with 100 mM H_2_O_2_ for 30 min were used as the positive control. The cells were fixed by 4% PFA for 30 min. These were then washed once with PBS and treated with PBS containing 0.3% TritonX-100 for 5 min at room temperature. Fifty μL of TUNEL detection solution was added to the cells, and they were incubated at 37 °C in the dark for 60 min. The cells were washed with PBS 3 times, and we observed the Cy3 excitation wave at 550 nm after mounting and image acquisition.

### 4.7. Cell Growth Curve

To obtain the growth curve of the cells, logarithmic phase cells with good growth states were harvested, and a cell suspension was made. The cell number was counted using a Nexcelom cellometer, and approximately 2.53 × 10^4^ cells were seeded into each well of 24-well culture plates. Three wells of cells were counted every 24 h, and the average number was calculated. Cell counting persisted for 8 days. Culture time was used as the horizontal axis, and the average number of cells per day was used as the vertical axis to plot the growth curve of cells.

### 4.8. Karyotype Analysis

The procedure of Chang et al. [21] was modified for karyotype analysis. Cells were cultured in a culture medium containing 0.02 µg/mL demecolcine for 2.5 h to arrest cells at M-phase. Then cells were trypsinized, pelleted by centrifugation and resuspended in a hypotonic solution containing 0.075 M KCl for 20 min, with gentle mixing every 5 min. A freshly prepared methanol-acetic acid solution (3:1) was used to fix the cells at room temperature for 40 min. Finally, resuspended cells were placed on pre-freezing slides. After drying, the slides were stained by Giemsa for chromosome staining. Images were captured using an inverted microscope (Nikon Ti-E).

### 4.9. Statistical Analysis

All experiments were performed with at least 3 replicates. All statistical analyses were carried out in GraphPad Prism software. The experimental data were analyzed, and the significant differences between treatments were calculated using a 1-way ANOVA in SPSS 22.0 statistical software [22]. Data are expressed shown as mean ± SEM, and *p*-values less than 0.05 were considered statistically significant.

## Data Availability

All original data will be provided upon reasonable request.
